# Whole‐mitochondrial genomes of *Nannizziopsis* provide insights in evolution and detection

**DOI:** 10.1002/ece3.9955

**Published:** 2023-03-27

**Authors:** Daniel Powell, Benjamin Schwessinger, Céline H. Frère

**Affiliations:** ^1^ Centre for Bioinnovation University of the Sunshine Coast Sippy Downs Queensland Australia; ^2^ School of Biological Sciences University of Queensland St Lucia Queensland Australia; ^3^ Research School of Biology The Australian National University Canberra Australian Capital Territory Australia

**Keywords:** diagnostic, fungal pathogen, mitochondrial genome, *Nannizziopsis*, qPCR, reptile

## Abstract

Infectious fungal diseases can have devastating effects on wildlife health and a detailed understanding of the evolution of related emerging fungal pathogen along with the ability to detect them in the wild is considered indispensable for effective management strategies. Several fungi from the genera *Nannizziopsis* and *Paranannizziopsis* are emerging pathogens of reptiles and have been observed to cause disease in a wide range of taxa. *Nannizziopsis barbatae* has become a particularly important pathogen of Australian reptiles with an increasing number of herpetofauna being reported with cases of infection from across the country. Here, we present the mitochondrial genome sequences and phylogenetic analysis for seven species in this group of fungi uncovering new information on the evolutionary relationship of these emerging pathogens. From this analysis, we designed a species‐specific qPCR assay for the rapid detection of *N. barbatae* and demonstrate its application in a wild urban population of a dragon lizard.

## Graphical abstract

In this article, we present the mitochondrial genome sequences and phylogenetic analysis for seven species of fungal pathogens from Nannizziopsis and Paranannizziopsis to provide insight into the evolutionary ecology of these emerging pathogens. In addition, we report a species‐specific qPCR assay for the rapid detection of the emerging reptile fungal pathogen Nannizziopsis barbatae and demonstrate its application in a wild urban reptile population experiencing an outbreak.

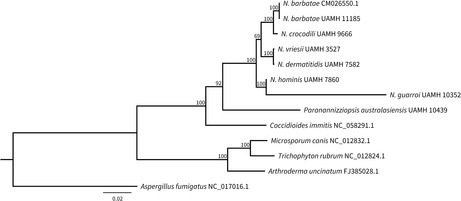

## 1 INTRODUCTION

Diseases‐causing fungi pose a serious threat to wildlife populations, yet despite the impact they have had on global biodiversity, they are notably understudied (Ghosh et al., 2018). New fungal pathogens are emerging that are capable of infecting an increasingly diverse range of taxa and their impacts are being exacerbated by changing climate conditions and globalization (Fisher et al., 2012; Ghosh et al., 2020). Urbanization can be a key driver of disease emergence, facilitating transmission due to newly overlapping geographic expansions (Hassell et al., 2017), increasing the risk of disease spillover from wildlife into humans and other animals (Heesterbeek et al., 2015). Reports of emerging fungal pathogens are rising (Fisher et al., 2020) and over the past few decades, a group of Onygenalean fungi from the genera *Nannizziopsis*, *Paranannizziopsis*, and *Ophidiomyces* have emerged as a leading cause of severe mycoses in reptiles (Paré & Sigler, 2016).

Fungi from the genus *Nannizziopsis* are capable of causing disease in several species of reptiles and have also been known to infect humans (Nourrisson et al., 2018). Infection in reptiles is contagious and initially presents as cutaneious disease with characteristic lesions forming crusts, ulcers, and hyperkeratosis that often progresses to fatal mycoses (Sigler et al., 2013). Among several species from this genus identified as reptile pathogens, infection with *Nannizziopsis barbatae* has become increasingly observed in free‐living populations of Australian reptiles with a wide variety of species being reported with this disease (Peterson et al., 2020). Urban wildlife populations in particular have become a focal point for outbreaks and the need for effective detection and monitoring of pathogen occurrence is considered vital for mitigating the spread and to minimize any potential for transmission to humans (Ghosh et al., 2018). Molecular diagnostic tests are powerful tools for disease surveillance offering a low cost and rapid means to assist in the early detection in both captive and wild populations (Boyle et al., 2004). Such tools also enable long‐term tracking of pathogens that facilitate the study of often complex host–pathogen interactions, such as how disease tolerance may effect prevalence and transmission (Seal et al., 2021; Tedersoo et al., 2019). Genomic data are valuable resources for the development of diagnostic tools enabling swift identification of target regions for designing highly specific markers, and these data can also serve as a foundation for studies on the molecular basis for pathogen evolution (DeCandia et al., 2018; Ghosh et al., 2020, 2021). Comparative genomic studies using whole‐mitochondrial genomes, as opposed to metabarcoding, can help to resolve evolutionary relationships by building more robust phylogenies in newly described species and thus may enhance the effectiveness of species‐specific detection methods (Tedersoo et al., 2019).

A rapid diagnostic tool to detect *N. barbatae* in clinical samples is currently unavailable. As *Nannizziopsis* fungi are typically first isolated on selective media prior to PCR and sequencing, laboratory diagnosis may involve delays of up to a week due to the slow growth of these species. The aim of this study is to gain a deeper understanding of the evolutionary relationship among this newly defined group of emerging fungal pathogens through the sequencing and analysis of their whole‐mitochondrial genomes and to develop a molecular diagnostic for the specific detection of *N. barbatae* infections to support the study of outbreaks of this disease in wild populations.

## 2 MATERIALS AND METHODS

### 2.1 Sample culturing and processing

Type cultures listed in Table 1 were purchased from the UAMH Centre for Global Microfungal Biodiversity in Toronto, Canada. *N. barbatae* strain USC001 was isolated and sequenced previously (Powell et al., 2021). Fungal cultures were grown on potato dextrose agar at 25°C for up to 3 weeks to obtain sufficient growth. Plate cultures were scraped, and DNA was purified using the DNeasy Plant Mini kit (Qiagen) with 5 mm Stainless Steel Beads (Qiagen). Purified DNA was then quantified and stored at −20°C. Short‐read sequencing was undertaken using the Nextera DNA Flex library kit and run on the Illumina NextSeq platform at Centre for the Analysis of Genome Evolution & Function at the University of Toronto. Skin swab samples (*n* = 96) were obtained from population of free‐living easter water dragons (*Intellagama lesueurii*) with a history of infection with *N. barbatae* in Brisbane city's Roma Street Parkland (−27°27′46′S, 153°1′11′E), Queensland, Australia. Skin lesions from animals displaying characteristic signs of infection were sampled with sterile rayon dry transfer swabs (Copan Diagnostics Inc.) and placed on ice before transportation to the laboratory to be stored at −20°C until processed to extract DNA using the Wizard Genomic DNA Purification Kit (Promega).

**TABLE 1:** Information and statistics for the mitochondrial genome assemblies for each fungal species.

Species	Strain	GenBank accession	Mitogenome size (bp)	GC %	Country of origin	Year isolated
*Nannizziopsis barbatae*	USC001	CM026550.1	30,107	23.7	Australia	2019
*Nannizziopsis barbatae*	UAMH 11185	ON968427	27,824	23.5	Australia	2009
*Nannizziopsis crocodili*	UAMH 9666	ON968429	30,841	23.7	Australia	1999
*Nannizziopsis vriesii*	UAMH 3527	ON968426	24,569	24.5	Netherlands	1972
*Nannizziopsis dermatitidis*	UAMH 7582	ON968430	25,568	24.2	Canada	1993
*Nannizziopsis hominis*	UAMH 7860	ON968431	30,161	23.8	USA	1994
*Nannizziopsis guarroi*	UAMH 10352	ON968428	30,493	23.6	USA	2003
*Paranannizziopsis australasiensis*	UAMH 10439	ON968432	31,311	23.4	Australia	2004

### 2.2 Assembly of mitochondrial genomes

Sequencing reads were trimmed and filtered for quality using Trimmomatic v0.36 (Bolger et al., 2014) before use with the GetOrganelle v1.7.3.5 tool (Jin et al., 2020) with the parameters ‐R 10 ‐k 21,45,65,85,105 ‐F fungus_mt for de novo assembly incorporating the SPAdes v3.13.0 assembler (Prjibelski et al., 2020) and Bowtie2 v2.4.2 (Langmead & Salzberg, 2012). Protein‐coding sequences were predicted using Mitos2 webserver (Donath et al., 2019) using the RefSeq 89 Fungi database. Coding sequences were manually inspected using Geneious Prime software v2022.2.1 (Biomatters Ltd.). Mitochondrial circular map was drawn using OGDRAW v1.3.1 (Greiner et al., 2019). Newly assembled mitochondrial genomes were submitted to the NCBI Genbank under accessions listed in Table 1.

### 2.3 Comparative analysis

A total of 13 protein coding genes (PCGs) could be consistently annotated across all genomes generated in this study and were chosen for phylogenetic inference. The PCGs *cox1*, *cox2*, *cox3*, *atp6*, *atp8*, *atp9*, *nad1*, *nad2*, *nad3*, *nad4*, *nad4L*, *nad5*, and *nad6* from 13 fungal strains were concatenated and aligned using MAFFT v7.221 (Katoh & Standley, 2013), and a small number of regions containing gaps were eliminated. PhyML v3.3.20180214 (Guindon et al., 2010) was used to construct the maximum‐likelihood tree using the LG method with 250 bootstrap replicates. The tree was visualized in Geneious Prime. Whole mitochondrial genome alignments were performed using Mauve v1.1.3 (Darling et al., 2010).

### 2.4 qPCR assay design

Extracted DNA from fungal cultures was checked with PCR using ITS gene fragment primers forward 5′‐GCATCGATGAAGAACGCAGCGA‐3′ and reverse 5′‐GGYCAGCKCCGGCCGGGTC‐3′ used in a previous study (Peterson et al., 2020) to confirm that purified DNA from the reference strains could be successfully amplified. Primers and probe were selected by manually examining nucleotide alignments of each coding sequence for regions of dissimilarity. The intronic region of the *N. barbatae* NADH dehydrogenase subunit I (*nad1*) gene was selected due to a larger number of polymorphisms compared with other genes (Figure 4). A custom primer and probe set was developed using the PrimerQuest™ Tool (Integrated DNA Technologies, Inc) and produced with the 6‐FAM five‐prime reporter dye and the ZEN/Iowa Black FQ double‐quenched probe system (Integrated DNA Technologies, Inc). The qPCR reactions consisted of 10 μL SensiFAST Probe No‐ROX Kit (Meridian Bioscience), 0.4 μM each of forward primer 5′‐TGATCATGTTTAGTCTCTGAAGGT‐3′ and reverse primer 5′‐AGGCTAAGCTGATTTGGTCTC‐3′, 0.1 μM of the probe 5′‐6‐FAM/TCCCTGCTG/ZEN/ATTGCCATATATTAGGT/FQ/−3′, and 2 μL DNA template made up to a volume of 20 μL using molecular grade water. The cycling parameters included an initial denaturation step of 95°C for 5 min followed by 35–40 cycles of 95°C for 20 s and 63°C for 30 s using a Rotor‐Gene Q HRM 5plex Platform (Qiagen). The resulting 114 bp amplicon was confirmed as the correct region using Sanger sequencing. A positive result was called for any sample crossing the cycle threshold (*C*_t_) as determined by the Rotor‐Gene Q software. Each qPCR assay was run with positive and negative controls in duplicate.

## 3 RESULTS

### 3.1 Mitochondrial genome assembly and phylogenetics

**FIGURE 1:**
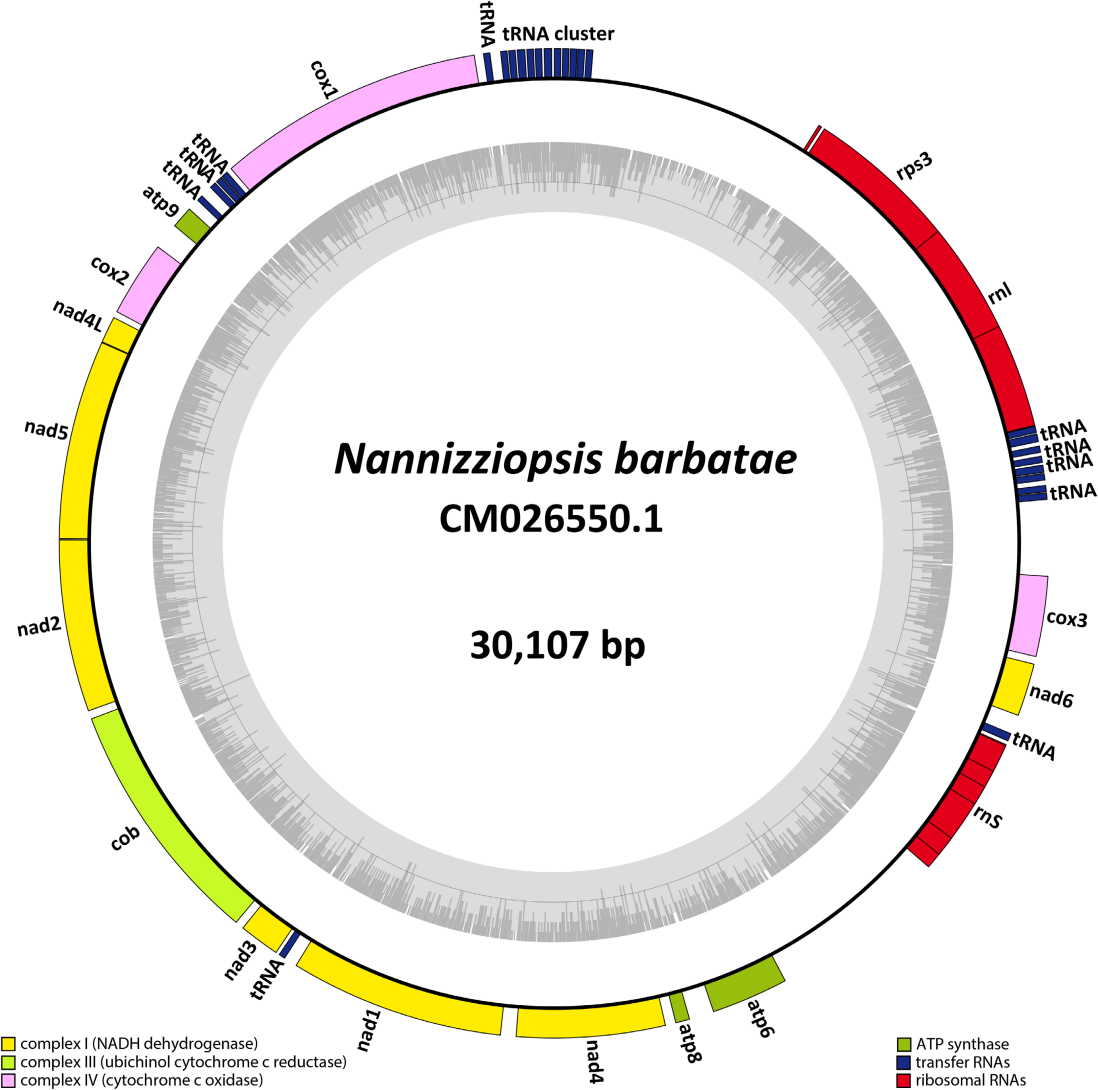
Circular representation of the mitochondrial genome of *Nannizziopsis barbatae* strain USC001 isolated from an infected Eastern water dragon. Protein coding genes are represented by colored bars. GC content is represented in the internal circle.

Short‐read shotgun sequencing produced a minimum 150‐fold read coverage per sample that was used to reconstruct the mitochondrial genomes from six *Nannizziopsis* and one *Paranannizziopsis* reference strains. Variability in mitochondrial DNA length between species ranged from 24.5 to 30.8 Kb (Table 1). The overall GC content was fairly consistent among species ranging between 23.4% and 24.5%, consistent with the AT‐rich composition of fungal mitochondrial genomes. The *N. barbatae* mtDNA is gene dense and contains the same gene order reported for other Onygenaceae (Figure 1; de Melo Teixeira et al., 2021).

**FIGURE 2:**
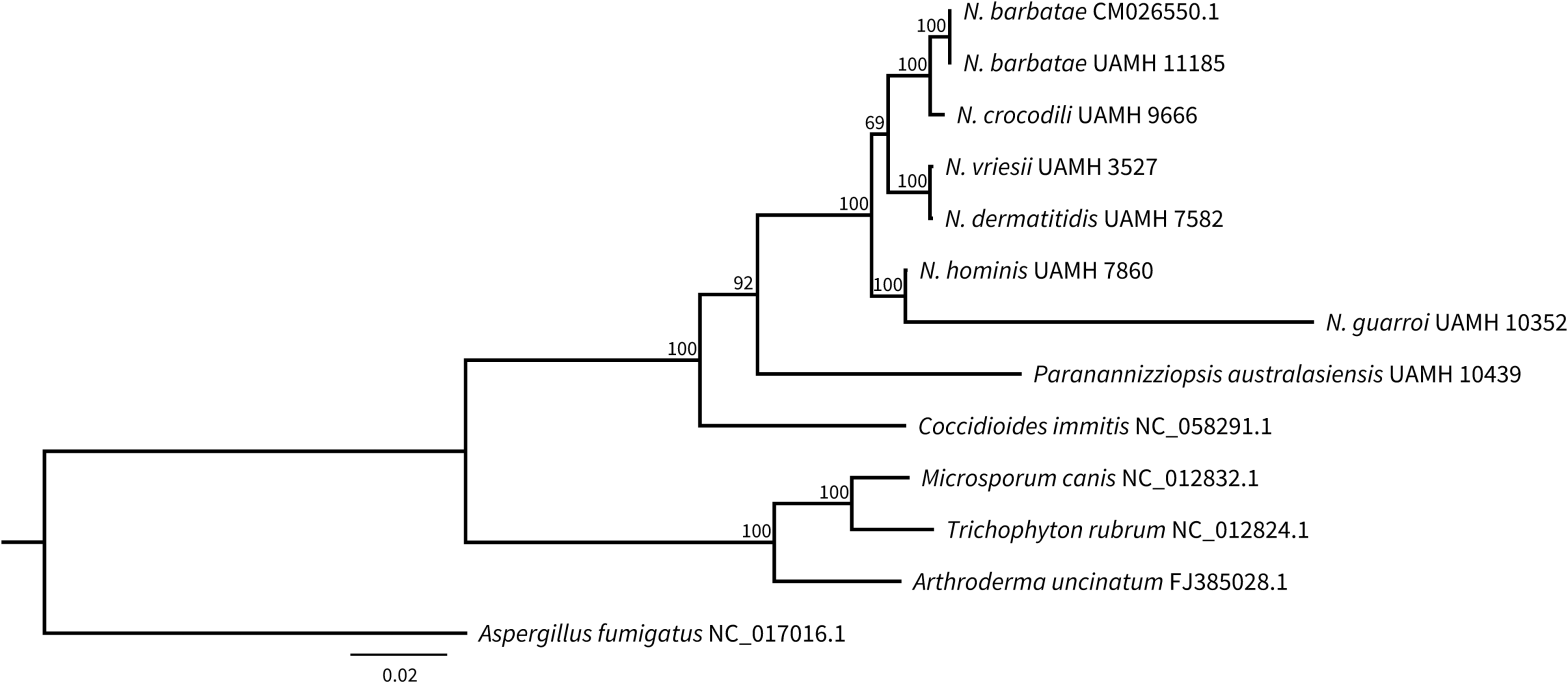
Maximum‐likelihood phylogenetic tree showing the evolutionary relationship between the seven mitochondrial genomes annotated in this study and with other species of onygenales fungi. *Aspergillus fumugatus*, a member of the Eurotiales, was used as the outgroup. The alignment contained 13 concatenated mitochondrial protein‐coding genes. Bootstrap support values obtained with 250 replicates are included at the nodes.

**FIGURE 3:**
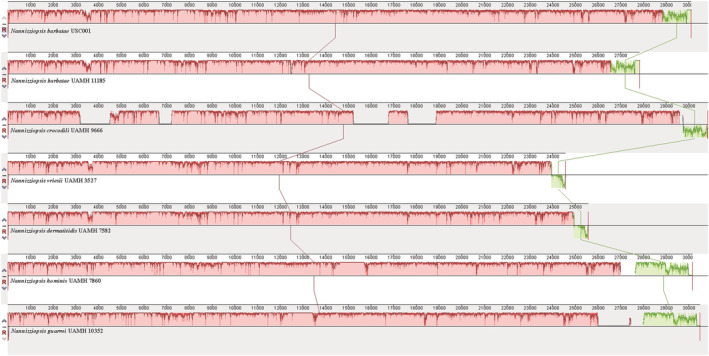
Analysis of collinearity of the seven *Nannizziopsis* fungi mitogenomes used in this study.

A total of 3780 positions were aligned across the 13 PCGs annotated for each of the genomes used in this study. Topology of the *Nannizziopsis* branches in the phylogenetic tree (Figure 2) resemble results from previous studies (Peterson et al., 2020; Sigler et al., 2013) showing congruence between the approach used in this study and the conserved nucleotide targets of the ITS and 28S ribosomal regions. Only two out of the 3757 aligned positions observed between the assemblies of *N. barbatae* were different suggesting a high level of conservation in PCGs between these strains. Likewise, only two out of the 3760 aligned amino acids between *N. vriesii* and *N. dermatiditis* were found to be different. Given the high degree of sequence conservation observed between these two pairwise comparisons, whole mtDNA alignments and analysis of collinearity were performed for all of the *Nannizziopsis* isolates to determine the degree of similarity at the nucleotide level (Figure 3). Within‐species whole mtDNA comparisons between *N. barbatae* strains USC001 and UAHM 11185 resulted in 1675 differences being observed among 27,757 aligned nucleotide positions (93.97% identical). However, the alignment between *N. vriesii* and *N. dermatiditis* resulted in only 154 differences out of 24,572 aligned nucleotide positions (99.37% identical). The *N. barbatae* strain USC001 assembly is slightly larger than the UAHM 11185 strain owing to the inclusion of two introns, one in the *cox1* gene and another in the large ribosomal subunit RNA gene (*rnl*) gene of approximately 1 kb each in size. The *cox1* intron was found to contain an LAGLI‐DADG endonuclease motif and the *rnl* intron an GIY‐YIG endonuclease motif. The smaller assembly size for *N. vriesii* when compared with *N. dermatiditis* is due to the former missing an approximately 1 kb intron in the *nad1* gene. This intron also contains an GIY‐YIG endonuclease motif.

### 3.2 Species‐specific qPCR performance

**FIGURE 4:**
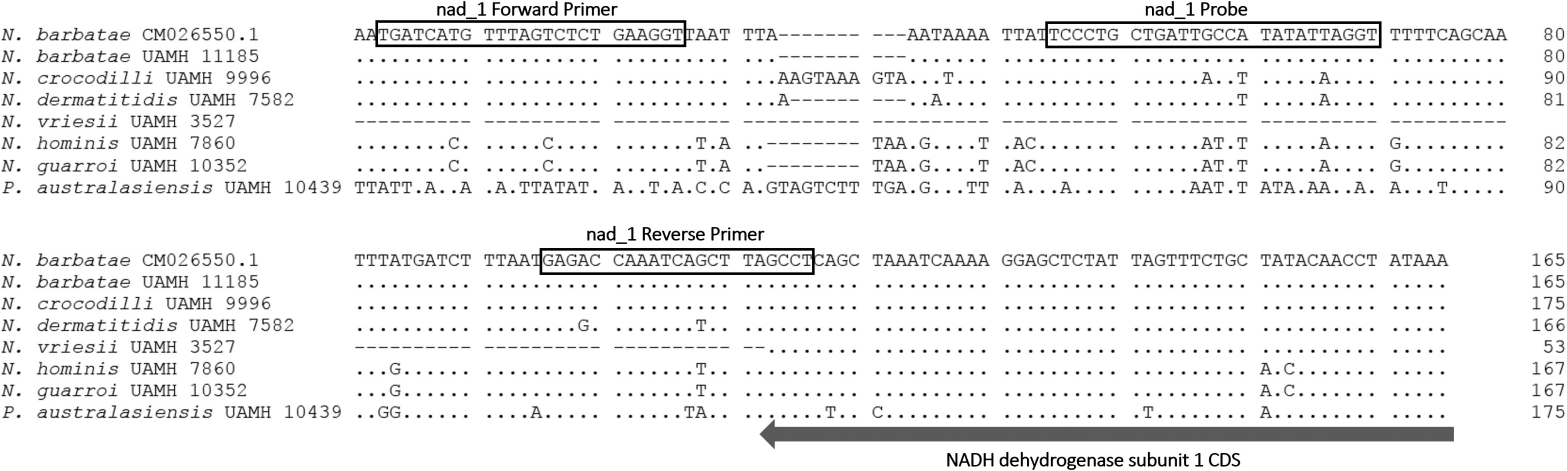
Fungal mtDNA sequence alignment of the *nad1* gene showing positions of the *Nannizziopsis barbatae*‐specific primers and probe used in this study. Dots indicate bases identical to the *N. barbatae* CM026550.1 reference sequence. The assay produces an amplicon 114 bp in length.

Using our qPCR assay, DNA from two different isolates of *N. barbatae* was successfully detected. Yet we could not detect any other *Nannizziopsis* species in this study, including the closely related *N. crocodili*. These results suggest that mtDNA targets can be used to distinguish between species of *Nannizziopsis* fungi. A small degree of non‐specific fluorescence was observed from *N. dermatitidis* DNA at the optimal probe annealing T_m_ of 60°C owing to a lower amount of variation in this species at the probe target location (Figure 4). This was resolved without sacrificing reaction efficiency by raising the annealing temperature to 63°C. The probe sequence confers the specificity in the assay as the PCR primer sequences were conserved across *Nannizziopsis* species and could produce an amplicon from all but *N. vriesii* (Figure S1) owing to the absence of the intron in the *nad1* gene in this species as mentioned above. Specificity of the assay to distinguish among the species tested was 100%.

**FIGURE 5:**
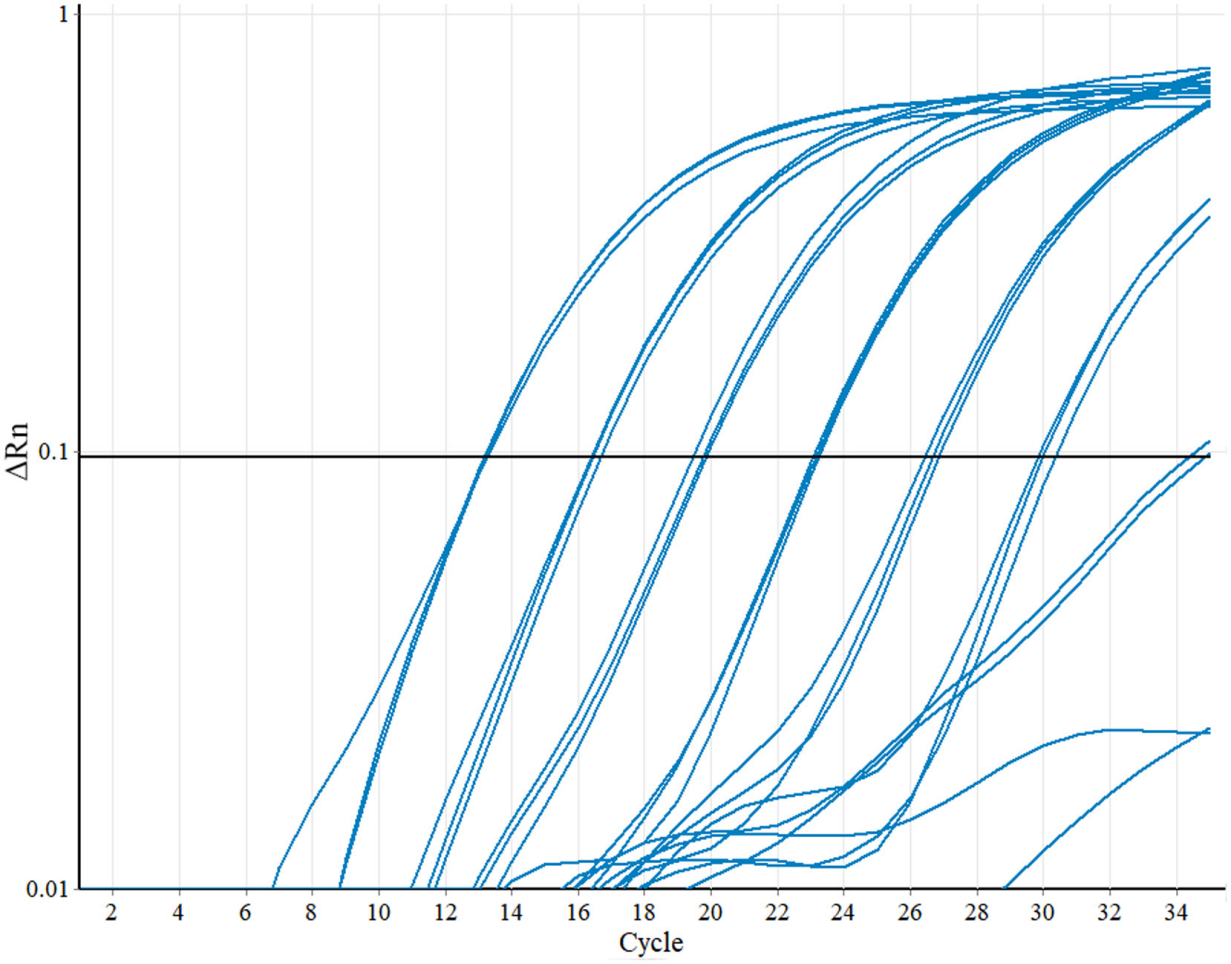
Amplification plot of the *Nannizziopsis barbatae*‐specific qPCR assay fluorescence values showing performance of purified *N. barbatae* DNA standards diluted over 7 orders of magnitude. Standard DNA was quantified and diluted across genome equivalent (GE) values 400,000, 40,000, 4000, 400, 40, 4, and 0.4 GE with each standard prepared in triplicate reactions.

Standard curve DNA was diluted across genome equivalent (GE) values 400,000 GE, 40,000 GE, 4000 GE, 400 GE, 40 GE, 4 GE, and 0.4 GE with each standard prepared in triplicate reactions in two separate qPCR runs to evaluate for reproducibility (Figure 5). The reaction efficiency ranged from 0.89 to 1.01, and the *R* ^*2*^ values were greater than .99 for both runs (ranging from 0.996 to 0.999). The *C*_t_ values of the quantitation standards were also consistent between runs with each dilution crossing the cycle threshold at approximately the same cycle number. Sensitivity of the assay was below 1 GE per reaction.

To test the performance of this assay to detect the presence of *N. barbatae* from field collected samples, a total of 96 skin swab samples were obtained from 85 individual animals that were either healthy or displaying various levels of disease severity (see example in Figure S2) over the course of 2021 and tested using this protocol. A total of 67 swab samples returned a positive result for infection with *N. barbatae* (Table 2). The *C*t values for each of the positive results ranged between 17 and 33. In each assay, the nontemplate controls did not cross the threshold.

**TABLE 2:** Summary of qPCR assay screening of skin swabs from visibly diseased or otherwise healthy dragons accompanied with assigned disease severity rating.

Disease rating	qPCR positive	qPCR negative	Sample count
1. Mild	33	6	39
2. Mild–moderate	13	1	14
3. Moderate	11	0	11
4. Moderate–severe	9	0	9
5. Severe	1	0	1
0. No obvious lesions	0	22	22
Total	67	29	96

## 4 DISCUSSION

In this study, we resolve the evolutionary relationship between key members of the *Nannizziopsis* responsible for diseases in reptiles using full‐length protein sequences of 13 mitochondrial protein coding genes. We report that the high similarity between the species *N. vriesii* and *N. dermatitidis* extends across the entire mitochondrial genome with the notable exception of an intron present in the *nad1* gene in *N. dermatitidis*. This high level of similarity suggests some taxonomic revision may be appropriate to either group these two species together or split the two strains of *N. barbatae*. The varying occurrence of introns was also observed between the two strains of *N. barbatae* in this study isolated from infected reptiles approximately 10 years apart. Each of these introns was found to contain either an LAGLI‐DADG or GIY‐YIG endonuclease domain motif. These domains encode homing nucleases, suggesting these introns possess a capacity for self‐splicing (Megarioti & Kouvelis, 2020). Intraspecific variations in the presence of mitochondrial introns have been reported for other species of fungi (Deng et al., 2020; Freel et al., 2014), and the nonuniform inclusion of introns in the mitochondrial genes found in these closely related species may indicate the activity of mobile elements during their evolutionary history. An intron is present in the *nad1* gene of all *Nannizziopsis* and *Paranannizziopsis* isolates included in this study with the exception of *N. vriesii* (UAMH 3527), which was originally isolated in 1972, two decades earlier than any of the other fungal species. This strain of *N. vriesii* was also found in Europe, quite distant from each of the other strains that were found in either Australia or North America. For each *N. barbatae* isolate, this intron is 1047 bp in length and was dissimilar in only two positions, which are outside of the assay target region, despite UAMH 1185 being isolated from its reptile host over 10 years prior and from a geographic location separated by more than 700 km. Even though the *nad1* gene intron contains an GIY‐YIG motif, it is conserved in all but one species of *Nannizziopsis* included in this study and could be amplified using the same primer pair in each case. We believe this DNA target is stable enough for further applications using this assay for the detection of *N. barbatae*.

This study furthermore offers a rapid method for the detection of *N. barbatae* DNA from cultured and field collected samples. Diagnostic confirmation had previously relied on cultivation and subsequent metabarcoding analysis to confirm presence of *Nannizziopsis* infection (Peterson et al., 2020) leading to lengthy delays in obtaining results due to the slow growth characteristics of these species (Paré & Sigler, 2016). Our qPCR assay was able to correctly distinguish DNA samples of *N. barbatae* from five other species of *Nannizziopsis*. The assay was sufficiently sensitive to detect the presence of less than one genomic equivalent per reaction, owing to the presence of multiple copies of the mitogenome for every nuclear genome. We expect there to be an average of between six to nine mitochondrial genome copies per nuclear genome for the *N. barbatae* isolates sequenced in this study based on differences observed in the amount of sequencing read coverage between the nuclear and mitochondrial genome. However, we caution that this may be highly variable and with only a few isolates sequenced to date, the use of higher limit of detection should be considered until sufficient data on copy number can be attained.

We also demonstrate the utility of this assay to screen a free‐living population of infected reptiles using minimally invasive swab sampling. In addition to the 67 samples that tested positive for infection with *N. barbatae*, there were seven instances of what appear to be false negative results out of 74 visibly diseased animals from this screening set. As these occurred only in the mildly diseased animals (rating 1 or 2, Table S1), we posit that this is likely the result of the state of the infected site being small and difficult to recover fungal DNA, and/or variation in the swab sampling techniques from different handlers that yielded lower sample material. There were no false positive results observed in this sample set suggesting no contaminating sources of nontarget DNA were encountered.

## AUTHOR CONTRIBUTIONS

**Daniel Powell:** Conceptualization (equal); data curation (equal); formal analysis (lead); methodology (lead); validation (lead); writing – original draft (lead). **Benjamin Schwessinger:** Data curation (equal); methodology (equal); writing – review and editing (equal). **Céline H. Frère:** Conceptualization (equal); funding acquisition (lead); resources (lead); writing – review and editing (equal).

## FUNDING INFORMATION

This work was funded by an Australian Research Council Future Fellowship (FT200100192) to CHF.

## CONFLICT OF INTEREST STATEMENT

The authors declare no conflicts of interest.

## Supporting information


Appendix S1

## Data Availability

The mitochondrial genome assemblies produced in this study are available from the NCBI GenBank under the accession numbers listed in Table 1. Primer sequences used to develop the assay are listed in the Section 2 (Materials and Methods).
