# Supplementary material for: Whole‐mitochondrial genomes of Nannizziopsis provide insights in evolution and detection
Source: Ecol Evol. 2023 Mar 27;13(3):e9955. doi: 10.1002/ece3.9955 (PMC10041364; doi:10.1002/ece3.9955)
Supplement: Supplementary file 1 — Appendix S1 [file ECE3-13-e9955-s001.docx]

A species-specific qPCR assay for the reptile pathogen *Nannizziopsis barbatae* and mitochondrial genomes of *Nannizziopsis* and *Paranannizziopsis*

Daniel Powell, Benjamin Schwessinger and Céline, H. Frère

**Supplementary Material**


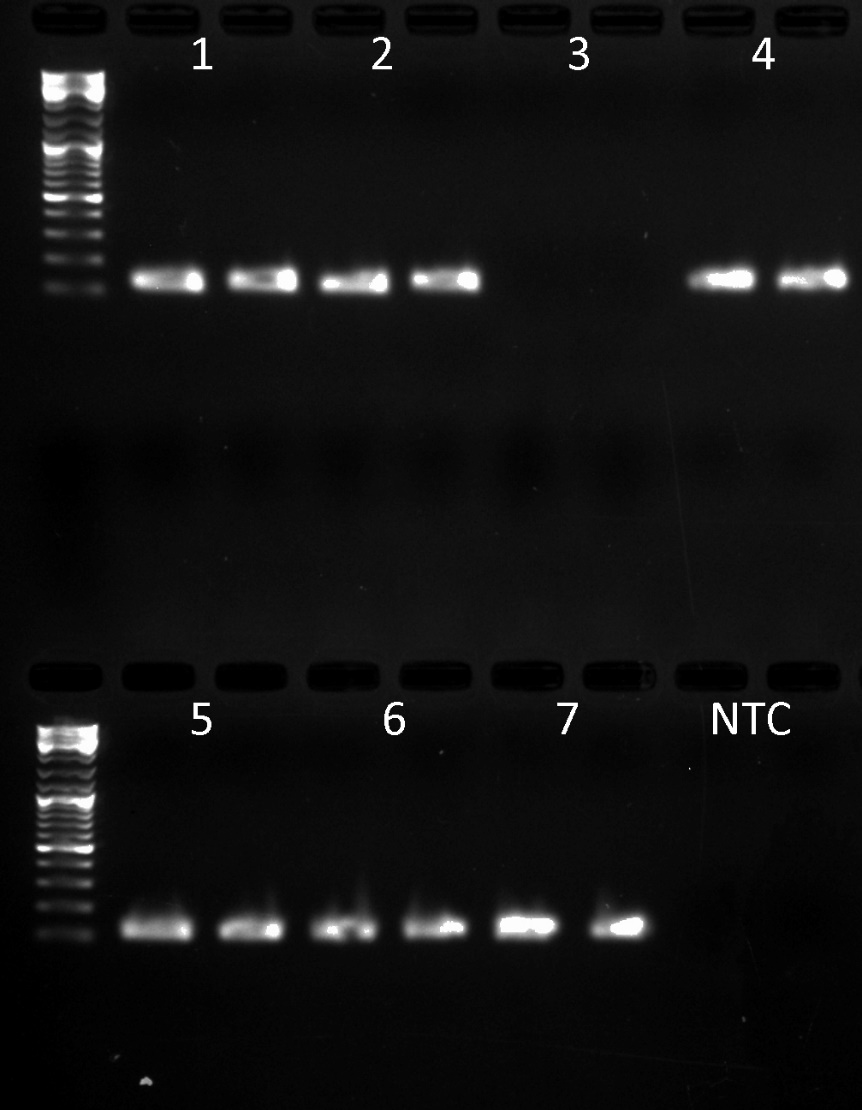


Supplementary Figure 1. Agarose gel electrophoresis image of fungal ITS PCR. DNA extracted from each of the reference cultures was subjected to PCR using the primers described in Materials and Methods. Each sample was run in duplicate reactions in the following order: 1, *N. barbate* UAMH 11185; 2, *N. crocodili* UAMH 9666; 3, *N. vriesii* UAMH 3527; 4, *N. dermatitidis* UAMH 7582; 5, *N. hominis* UAMH 7860; 6, *N. guarroi* UAMH 10352; 7, *P. australasiensis* UAMH 10439; NTC, non-template control.


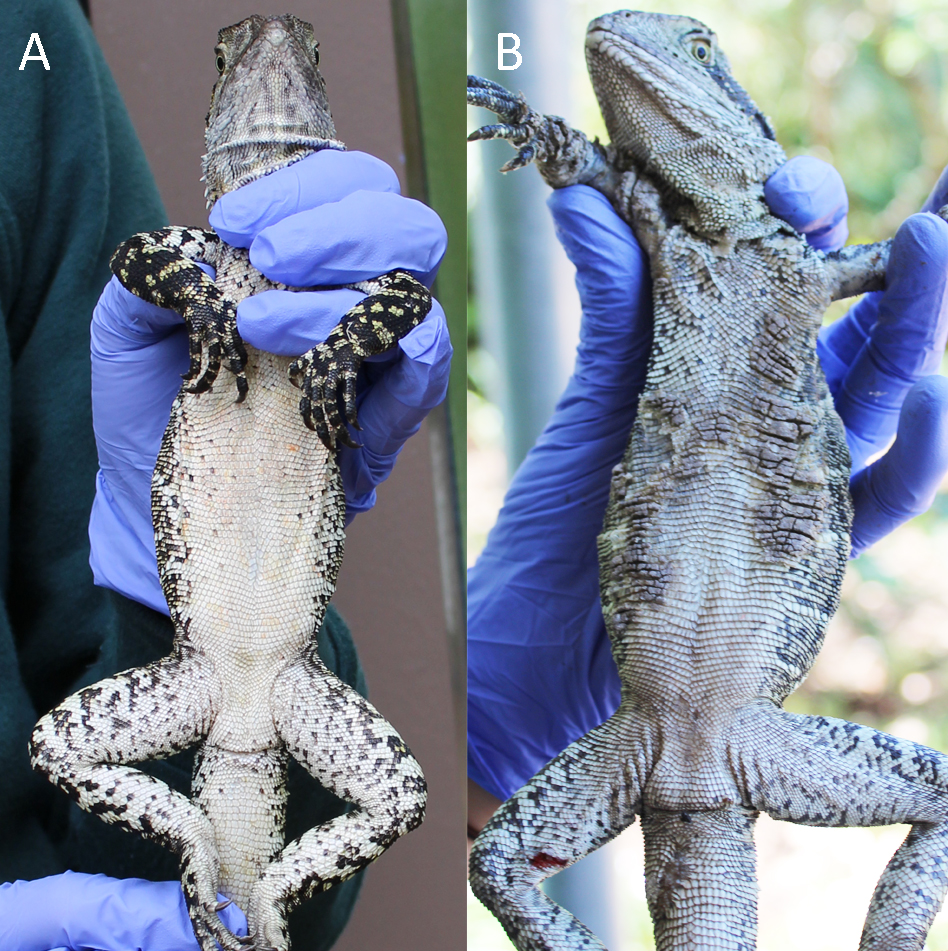


Supplementary Figure 2. Example of disease presentation in eastern water dragons. A) an example of a healthy female eastern water dragon. B) An example of a severely diseased female eastern water dragon with prominent crusting along the ventral skin surface and extremities.

Supplementary Table 1. Scale used to rate disease severity in eastern water dragons when caught in the field.

| **Rating** | **Degree of disease** | **Description** |
| --- | --- | --- |
| 0 | No obvious lesions | Normal, no skin lesions observed (e.g., only injury observed OR unsure of skin condition) |
| 1 | Mild | One to three focal skin lesions ≤ 5mm diameter |
|  |  |  |
| 2 | Mild/Moderate | Four to five focal skin lesions ≤ 5mm diameter, or one lesion 5-10mm in diameter |
|  |  |  |
| 3 | Moderate | Up to three lesions 5-10mm diameter, or one to two skin lesions 10-20mm in diameter |
|  |  |  |
| 4 | Moderate/Severe | Four+ skin lesions 10-20mm in diameter with roughly 5-10% of the skin surface affected |
|  |  |  |
| 5 | Severe | More than 10% of total skin surface affected, or 5-10% affected and in poor condition |
